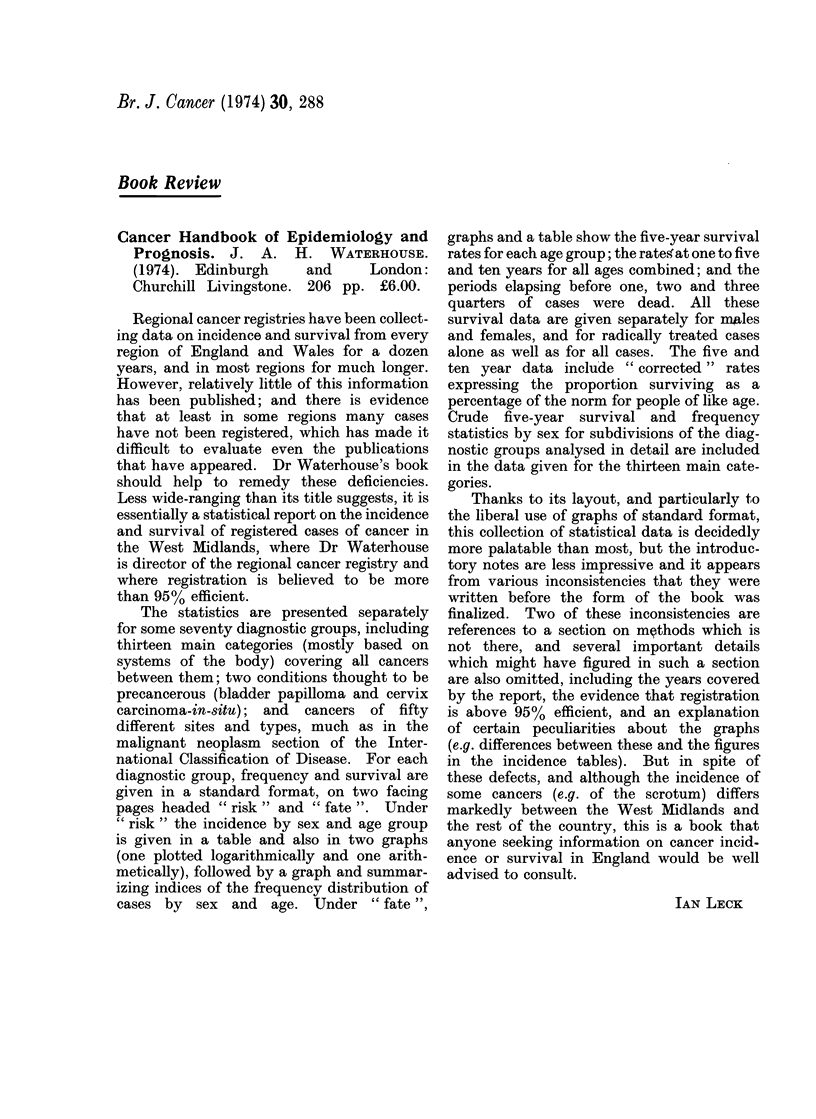# Cancer Handbook of Epidemiology and Prognosis

**Published:** 1974-09

**Authors:** Ian Leck


*Br. J. Cancer* (1974) **30**, 288

### *Book Review*

**Cancer Handbook of Epidemiology and Prognosis.** J. A. H. WATERHOUSE. (1974). Edinburgh and London: Churchill Livingstone. 206 pp. £6.00.

Regional cancer registries have been collecting data on incidence and survival from every region of England and Wales for a dozen years, and in most regions for much longer. However, relatively little of this information has been published; and there is evidence that at least in some regions many cases have not been registered, which has made it difficult to evaluate even the publications that have appeared. Dr Waterhouse's book should help to remedy these deficiencies. Less wide-ranging than its title suggests, it is essentially a statistical report on the incidence and survival of registered cases of cancer in the West Midlands, where Dr Waterhouse is director of the regional cancer registry and where registration is believed to be more than 95% efficient.

The statistics are presented separately for some seventy diagnostic groups, including thirteen main categories (mostly based on systems of the body) covering all cancers between them; two conditions thought to be precancerous (bladder papilloma and cervix carcinoma-*in-situ*); and cancers of fifty different sites and types, much as in the malignant neoplasm section of the International Classification of Disease. For each diagnostic group, frequency and survival are given in a standard format, on two facing pages headed " risk " and " fate ". Under " risk " the incidence by sex and age group is given in a table and also in two graphs (one plotted logarithmically and one arithmetically), followed by a graph and summarizing indices of the frequency distribution of cases by sex and age. Under " fate ", graphs and a table show the five-year survival rates for each age group; the rates at one to five and ten years for all ages combined; and the periods elapsing before one, two and three quarters of cases were dead. All these survival data are given separately for males and females, and for radically treated cases alone as well as for all cases. The five and ten year data include " corrected " rates expressing the proportion surviving as a percentage of the norm for people of like age. Crude five-year survival and frequency statistics by sex for subdivisions of the diagnostic groups analysed in detail are included in the data given for the thirteen main categories.

Thanks to its layout, and particularly to the liberal use of graphs of standard format, this collection of statistical data is decidedly more palatable than most, but the introductory notes are less impressive and it appears from various inconsistencies that they were written before the form of the book was finalized. Two of these inconsistencies are references to a section on methods which is not there, and several important details which might have figured in such a section are also omitted, including the years covered by the report, the evidence that registration is above 95% efficient, and an explanation of certain peculiarities about the graphs (*e.g.* differences between these and the figures in the incidence tables). But in spite of these defects, and although the incidence of some cancers (*e.g.* of the scrotum) differs markedly between the West Midlands and the rest of the country, this is a book that anyone seeking information on cancer incidence or survival in England would be well advised to consult.

IAN LECK